# The Art of Intercellular Wireless Communications: Exosomes in Heart Disease and Therapy

**DOI:** 10.3389/fcell.2019.00315

**Published:** 2019-12-03

**Authors:** Mallikarjun Patil, John Henderson, Hien Luong, Divya Annamalai, Gopalkrishna Sreejit, Prasanna Krishnamurthy

**Affiliations:** Department of Biomedical Engineering, Schools of Medicine and Engineering, University of Alabama at Birmingham, Birmingham, AL, United States

**Keywords:** exosomes, cardiac remodeling, immune modulation, diabetes, exosome engineering

## Abstract

Exosomes are nanoscale membrane-bound extracellular vesicles secreted by most eukaryotic cells in the body that facilitates intercellular communication. Exosomes carry several signaling biomolecules, including miRNA, proteins, enzymes, cell surface receptors, growth factors, cytokines and lipids that can modulate target cell biology and function. Due to these capabilities, exosomes have emerged as novel intercellular signaling mediators in both homeostasis and pathophysiological conditions. Recent studies document that exosomes (both circulating or released from heart tissue) have been actively involved in cardiac remodeling in response to stressors. Also, exosomes released from progenitor/stem cells have protective effects in heart diseases and shown to have regenerative potential in the heart. In this review we discuss- the critical role played by circulating exosomes released from various tissues and from cells within the heart in cardiac health; the gap in knowledge that needs to be addressed to promote future research; and exploitation of recent advances in exosome engineering to develop novel therapy.

## Introduction

Exosomes are bi-layered extracellular nanoscale vesicles released by almost all cells in the body that carry proteins, lipids, cytokines, transcription factors, and nucleic acids. Since their discovery three decades ago, exosomes have been implicated in various pathophysiological conditions. These extracellular vesicles were discovered back in the 80’s by two independent research groups investigating the fate of transferrin receptors in reticulocytes (Harding and Stahl, 1983; Pan and Johnstone, 1983). They observed that small vesicles were associated with transferrin receptors and were also released from reticulocytes to the extracellular fluid during recycling of the receptor. The work highlighted the mechanism by which reticulocytes lose these receptors during their maturation to erythrocytes (Harding et al., 2013). This was the first described biological phenomenon mediated by extracellular vesicles. These extracellular vesicles were later named exosomes (Pan and Johnstone, 1983). Exosomes were subsequently shown to be released from many cell types including dendritic cells (Zitvogel et al., 1998), macrophages (Ramachandra et al., 2010), B cells (Raposo et al., 1996), T cells (Blanchard et al., 2002), mast cells (Skokos et al., 2003), stem cells (Barile et al., 2014), and cancer cells (Taylor and Gercel-Taylor, 2008).

Exosomes measure between 30 and 100 nm and are secreted by cells in response to pathophysiological stimuli. They are actively synthesized by the endolysosome pathway and secreted into the intercellular space or into the systemic circulation. Exosomes were thought to be non-functional contents excreted from the cells in the form of vesicles, however, research in the recent past has shown that they function as intercellular communicators involved in cellular and organ cross talk. The cargo of exosomes contains lipids, proteins, micro-RNA, long non-coding (Lnc)-RNA, DNA and cell membrane receptors. Exosome membranes are made of a lipid bilayer and reflect the cells of origin; for example, exosomes from immune cells will have either MHC or T-cell receptor or B-cell receptor molecules whereas exosomes from cardiac progenitor cells express CD73/CD90/CD105 (Andriolo et al., 2018). However, the contents of exosomes are actively sorted and may or may not reflect the molecules from the cells of origin. For example, certain miRNA are selectively sorted into exosomes and these may potentially be absent in parent cells (Guduric-Fuchs et al., 2012; Zhang et al., 2015). Interestingly, these exosomes are taken up by the target cells through endocytosis mediated by cell adhesion molecules, specific receptors and specific membrane lipids or carbohydrates (Villarroya-Beltri et al., 2014). The contents of the exosomes can reprogram the target cells by activating specific signaling pathways via binding to specific receptors or through the delivery of enzymes and transcriptional regulators. Exosomal contents including micro-RNA have emerged as key regulators of target cell biology and function, thus exosomes are now recognized as a new class of paracrine signaling mediators in addition to classical pathways of intercellular communication by hormones, inflammatory mediators and cytokines. A detailed biology of exosomes is reviewed elsewhere (Villarroya-Beltri et al., 2014; Zhang et al., 2015; Ha et al., 2016). A present limitation of exosome research is that the tools required to identify the source and targets of exosomes in animals and humans are lacking, so cell-specific conclusions have instead been mostly been drawn from *in vitro* studies utilizing different cell types. Some of the tools that have been useful in understanding the biology of exosomes are summarized in Table 1.

**TABLE 1:** Common techniques and tools for understanding the biology of exosomes.

**Process**	**Tools**
Exosome biogenesis inhibition	GW4869, Manumycin A, Tipifarnib, Netoconizole, Ketoconozole, Climbazole, Dimethyl amiloride
Exosome uptake inhibition	Heparan, Cyclochalasin, Wortmannin, Cannabidiol
Genetic tools to label extracellular vesicle membranes	EGFP and dTomato tagged to N termini of palmitoylation signal
Genetic tools to label extracellular vesicle mRNA	Palm dTomato tagging to MS2 RNA sequences (bacteriophage MS2 coat protein)
Exosome RNA labeling	Syto RNA select
Endogenous labeling	NIR_AZA1 (BF2- azadipurromethene), DiR, DiD, Rlucm, 1,1’-Dioctadecyl-3,3,3’,3’-Tetramethylindotricarbocyanine Iodide, PKH, cy7
Bioluminescence labeling	firefly luciferase, D-luciferin, Renilla luciferase, gaussia luciferase
Visualization	Electron microscopy, internal reflection fluorescent microscopy, single molecule localization microscope
Nanoparticle analysis and characterization	Nanosight, dynamic light scattering

The published data suggests that exosomes play critical roles in the patho-physiology of heart diseases; and have been increasingly investigated for their potential use in diagnosis and therapy due to their unique size, structure, and composition. In this review, we provide an overview of the role of exosomes in intercellular signaling and its effects on heart disease; the influence of various organ systems and co-morbidities including cardiometabolic syndromes on the pathogenesis of exosome-mediated heart disease; and bioengineering exosomes for efficient therapy.

## Exosomes in Heart Disease

**FIGURE 1:**
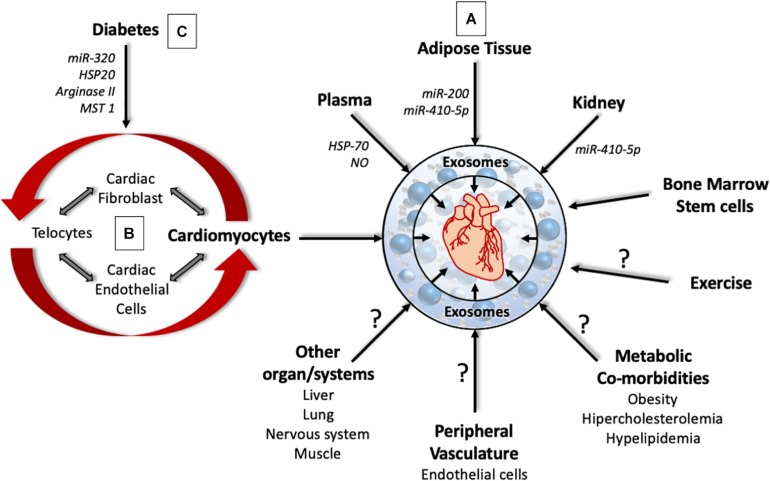
The complex role of cardiac and non-cardiac derived exosomes in cardiac pathophysiology. **(A)** Exosomes from different organs affect the pathophysiology of the heart through the delivery of signaling molecules, micro-RNA and enzymes. However, the role of exosomes derived from the liver, lung, muscle, nervous system, metabolic co-morbidities, and exercise that can affect heart health are yet to be determined. **(B)** Exosomes from different cell types within the heart can program cells within the heart to promote cardiac remodeling that affect heart structure and function. **(C)** Under Diabetic milieu, exosomes from different cell types within the heart can bring about coronary endothelial dysfunction, cardiac hypertrophy, and cardiac fibrosis.

Heart diseases are the leading cause of morbidity and mortality worldwide. According to the Center for Disease Control (CDC), 1 in 4 deaths are attributed to heart diseases in the United States and the economic burden of heart disease is estimated to be $300 billion annually (Benjamin et al., 2019). Cardiac structure and function in heart disease is regulated through communication between different cell types within the heart (fibroblasts, endothelial cells, cardiomyocytes, and Telocytes) and between the heart and peripheral tissues/organs like vasculature, kidney, bone marrow, lungs, and immune cells. Studies have shown that exosomes participate in the organ/tissue cross talk and play a critical role in the pathogenesis of heart diseases, including myocardial infarction (MI) (Figure 1). Extensive research has shown that exosomes can very well be utilized for diagnosis and treatment of cardiac diseases summarized in Table 2, and discussed below; therefore, a better understanding of the biological functions surrounding exosomes in the context of cardiac pathophysiology is necessary for the development of novel therapies.

**TABLE 2:** Studies demonstrating exosomes diagnosis and treatment of heart diseases.

**Functional component**	**Disease**	**Species**	**References**
miR-126 and miR-26	CAD	Humans	Zampetaki et al., 2010
miR-223, miR-29b RNU6-2	CV risk in smokers	Humans	Badrnya et al., 2017
miR-1, miR-21, miR-133, miR-146a, miR-208b, miR-499	Ischemic heart disease	porcine	Deddens et al., 2016
Apolipoprotein C-III, Apolipoprotein D, complement C1q subcomponent A, platelet glycoprotein 1b alpha chain, platelet basic protein	Ischemic Heart Disease	Humans	Cheow et al., 2016
miR-194, miR-34	Heart failure post-MI	Humans	Matsumoto et al., 2013
miR-146a	Peripartum cardiomyopathy	Humans	Halkein et al., 2013
miR-17, miR-197, miR-509-5p, miR-92a, miR-320a,	Metabolic syndrome	Humans	Karolina et al., 2012
miR-1, miR-133a, miR-208	Acute coronary Syndrome	Humans	Kuwabara et al., 2011
CD144 EMP	Coronary heart disease	Humans	Nozaki et al., 2009
Endothelial microparticle	Cardiovascular mortality in end stage renal failure	Humans	Amabile et al., 2010
Endothelial microparticle	Preeclampsia	Humans	González-Quintero et al., 2003
miR-126 and MiR-199a	Cardiovascular events	Humans	Jansen et al., 2014
CCL2, CCL7 and IL6	Cardiac inflammation post-MI	Mouse Humans	Loyer et al., 2018
Inflammatory and immunoglobulin class proteins	Transplant rejection	Humans	Kennel et al., 2018
Cardiac bridging integrator 1	Heart failure	Humans	Nikolova et al., 2018
miR-92b-5p	Dilated cardiomyopathy	Humans	Wu et al., 2018, p. 199
miR-223	Sepsis (microbial and non-microbial)	Mouse	Wang et al., 2015

## Exosomes in Diabetic Cardiomyopathy

Diabetes affects over half a billion people all around the world, cardiovascular diseases are the major cause of morbidity and mortality in diabetes, with more than half of the deaths in older diabetic patients being directly attributed to cardiovascular diseases (Donahoe et al., 2007; Matheus et al., 2013). Diabetic patients are at a higher risk of developing cardiovascular diseases compared to non-diabetic patients due to the damage caused to blood vessels, inhibition of angiogenesis, increased inflammation, metabolic changes in cardiac cells, cardiomyocyte death and defective phagocytosis, which can lead to cardiac fibrosis, hypertrophy, and cardiac stiffness (Miki et al., 2013; Suresh Babu et al., 2016; Jia et al., 2018). While the interplay of diabetes and heart disease is highly complex, new evidence show that exosomes play an important role in the pathology. In diabetes, exosome cargo and structural composition are modified as the parent cells are affected by the diabetic milieu (Kamalden et al., 2017; Bellin et al., 2019). Exosomes in diabetic milieu have been shown to inhibit angiogenesis, promote cell death, and induce due to loading of maladaptive contents that affect transcription and signaling pathways (Sahoo and Emanueli, 2016; Tao et al., 2019). Consistent with these reports, circulating exosomes in diabetics were low in proangiogenic factors like miR-126 and miR-26 (Zampetaki et al., 2010). In addition, cardiomyocyte derived exosomes under diabetic condition exhibited over expression of anti-angiogenic factor miR-320 (Wang et al., 2014). Co-culture experiments utilizing cardiomyocytes from diabetic rats and mouse cardiac endothelial cells inhibited proliferation and migration of mouse endocardial epithelial cells (MCEC). Interestingly, the endothelial cell dysfunction was abrogated by treatment with GW4869, an inhibitor of exosome formation and release, suggesting that exosomes from diabetic cardiomyocytes were responsible for myocardial endothelial cell dysfunction (Wang et al., 2014). Consistent with this finding, treatment of MCEC cells with exosomes from Goko Kakizaki rat (diabetic) cardiomyocytes inhibited endothelial cell proliferation, migration and tube formation. Mechanistically, these effects were shown to be mediated by exosome derived miR-320 from diabetic rats, which is shown to target Insulin like growth factor 1 (IGF1), HSP 20, and Ets2, important mediators of endothelial cell function. Also, transgenic mice overexpressing HSP20 in cardiomyocytes, protected mice against diabetic cardiomyopathy. In co-culture experiments, cardiomyocytes overexpressing HSP20 promote endothelial cell proliferation, migration and tube formation (Wang et al., 2016; Davidson et al., 2018a). Exosomes from HSP-Tg mice (transgenic mice overexpressing HSP20) had higher levels of phosphorylated Akt, survivin and super oxide dismutase, that are shown to protect against diabetes mediated-oxidative stress in endothelial cells and cardiomyocytes. Furthermore, cardiac pathology mediated by exosomal HSP70 in diabetes was rescued by the addition of exosomes from healthy rats. Systematically, exosomes from healthy mice activate TLR4, which in turn protects cardiomyocytes through ERK1/2 and HSP27 (Davidson et al., 2018a).

Diabetes induced endothelial dysfunction has also been known to be mediated by exosomes. In diabetes exosomes are enriched with arginase 1 and these exosomes were shown to inhibit ACE-induced endothelin dependent relaxation in aortas. This pathology was inhibited by heparin treatment, suggesting primary contributor to be exosomes (Zhang et al., 2018). It was found that the enriched Arginase1 compromised the availability of L-arginine, impairing nitric oxide (NO) production leading to defective aortic relaxation.

Exercise is known to have beneficial effects on the health of diabetic patients, especially in cardiovascular health. Several mechanisms have been proposed, including production of cardioprotective exosomes. A recent study by Chaturvedi et al. has shown that exercise enriches specific cardioprotective miRNA’s such as miR-455, miR-323, miR-466, and miR-29b. Mechanistically, the cardio-protective effect was primarily due to the inhibition of MMP9, a matrix protein primarily involved in cardiac fibrosis. The expression of MMP9 was inhibited at the transcription level by miR-29b and miR-455 by the binding of its 3 prime UTR (Chaturvedi et al., 2015).

In summary, the published data suggests that exosomes under diabetic milieu carry miRNA, proteins and enzymes that are involved in the pathogenesis of cardiomyopathy. Therefore, future studies are necessary to investigate whether exosome biology/function can be re-programmed to promote cardiac health, which could be therapeutically valuable for the treatment of diabetes.

## Adipocyte-Derived Exosomes Affecting Cardiac Health

Adipose tissue was once thought to be an energy storage organ, however, adipose tissue has emerged as an endocrine organ secreting hormones, paracrine factors and inflammatory mediators. Recent studies suggest that adipocytes also secrete exosomes that play a critical role in obesity mediated metabolic disorders (Ying et al., 2017; Zhao et al., 2018). Interestingly, the majority of circulating exosomes are derived from adipose tissue and their content is determined by the health of the adipose tissue (Barberio et al., 2019). Adipose tissue biology is primarily regulated by the transcription factor PPAR-gamma and its agonists have been used for insulin sensitization in diabetic patients. However, PPAR-gamma agonists (rosiglitazone) cause heart failures that may be mediated by exosomes from adipose tissue. In co-culture experiments, rosiglitazone treated adipocytes induced cardiomyocyte hypertrophy, however, this was inhibited by treatment with GW9662 (rosiglitazone inhibitor) and GW-4869 (exosome biogenesis inhibitors), suggesting that the induction of hypertrophy was due to PPAR-gamma signaling mediated by exosomes. Molecular analysis showed that PPAR-gamma induced miR-200 expression in adipocytes and this was enriched in exosomes. miR-200 enriched exosomes induced cardiac hypertrophy by inhibiting tuberous sclerosis, the negative regulator of mTOR (Fang et al., 2016).

Adipose tissue derived exosomes have also been implicated in the development of atherosclerosis, a risk factor for heart disease. Atherosclerosis is characterized by plaque formation in the arteries that can lead to the narrowing of arteries that limits blood supply. An important aspect of atherosclerosis is the infiltration of inflammatory foam cell macrophages that take up oxidized low-density lipoproteins. Interestingly, the injection of exosomes from visceral fat explants of obese mice exacerbated atherosclerosis in mouse models of the disease. Mechanistically, exosomes from obese visceral fat explants induced the formation of M1 macrophages through the activation of NFkB pathway in RAW 264.7 cells (Xie et al., 2018). This data suggests that adipose tissue derived exosomes can reprogram macrophages into pro-inflammatory M_1_ phenotype cells thus further increasing the potential for macrophage infiltration and chronic atherosclerotic inflammation.

## Immune Cells-Derived Exosomes and Its Role in Cardiac Pathophysiology

The immune system is primarily involved in the body’s defense against pathogens, wound repair, inflammation and protection against non-self-antigens. Recent evidence has shown that exosomes play important role in carrying out these functions (Qazi et al., 2009; Lugini et al., 2012). Immune cells play an active role post-MI in the heart by clearing out cell debris, resolve inflammation and facilitating cardiac remodeling (Anzai et al., 2012; Hofmann et al., 2012). Consistent with this, depletion of dendritic cells resulted in sustained inflammation, inhibited endothelial cell proliferation, and thus resulting in worse outcomes (cardiac functions and cardiac remodeling), post-MI (Anzai et al., 2012). Interestingly, exosomes mediate some of these effects. For example, exosomes from cardiomyocytes exposed to ischemia or necrosis stimulate differentiation of bone marrow derived dendritic cells, which in turn secrete exosomes that program splenic CD4^+^ T cells to secrete chemokines and inflammatory mediators; improving cardiac function post-MI (Liu et al., 2016). This suggests cardiomyocytes exposed to hypoxic conditions release exosomes that mobilize immune cells to the infarct zone and promote cardiac health. Similarly, macrophage derived exosomes enriched in miR-155 inhibited cardiac fibroblast proliferation post-MI in mice through the inhibition of son of Sevenless 1, a critical modulator of RAS activation. Macrophage derived miR-155 also promote cardiac inflammation by increasing the secretion of inflammatory cytokines IL1β, IL-6, TNF-alpha, and CCL2. Moreover, miR-155 knockout mice hearts were protected from adverse effects post-MI as shown by cardiac fibroblast proliferation and protection from cardiac rupture (Wang et al., 2017). Overall, this data suggests that immune cells orchestrate several events post-MI to promote the clearing of debris, inflammation and wound healing by distinct mechanisms that utilize exosomes.

**FIGURE 2:**
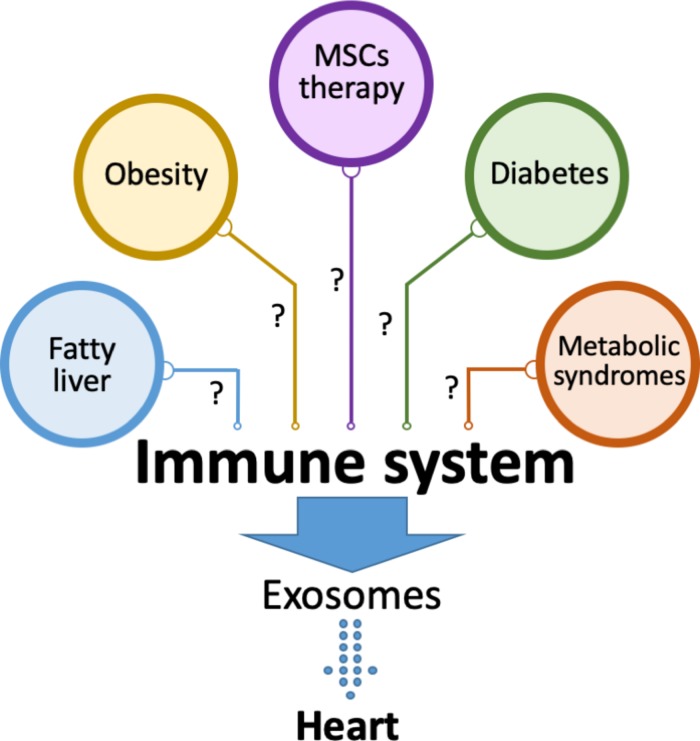
Exosomes from the immune system affect cardiac tissue. Immune system is modulated under different metabolic settings including diabetes, obesity, fatty liver and metabolic syndromes. The role of exosomes secreted by immune cells under these conditions in heart pathology is yet to be fully described. It is also not known whether the beneficial effects of stem cell-based therapies in heart diseases are mediated by immune modulation that may lead to the release of cardio protective exosomes.

While data on exosomes from immune cells and their effects on heart disease is evolving, studies point out to a high incidence and poor outcome of heart diseases in different metabolic settings (diabetes and obesity) in the absence of other risk factors. These differences could be explained in part by exosomes derived from immune cells, given the fact that the immune system is reprogrammed under metabolic milieu. For example, the immune system is compromised in diabetes and can lead to defective wound repair and increased inflammation. Consistent with this notion, research from our lab has shown that exposure to high glucose decreases the expression of miR-126 in macrophages and thus decreases the ability of macrophages to clear apoptotic cardiomyocytes. Moreover, overexpression of miR-126 rescued the functional deficiency of macrophages (Suresh Babu et al., 2016). In addition, Gao et al. reported that exosomes from mature dendritic cells promote atherosclerosis by inducing endothelial inflammation through exosome derived tumor necrosis factor-alpha (TNF-alpha), which activates the pro-inflammatory NF-kB pathway (Gao et al., 2016). Moreover, macrophage-derived exosomes promote the formation and mineralization of atherosclerotic plaques (Xie et al., 2018). Therefore, it is logical to speculate that the outcomes of heart diseases under metabolic conditions could be mediated by exosomes from immune cells exposed to metabolic milieu (Figure 2). In addition, stem cell/progenitor cell therapy can improve cardiac health post-MI through the use of exosomes (Khan et al., 2015; Kishore and Khan, 2016), however the mechanisms have yet to be fully worked out. Therefore, future research is necessary to investigate whether stem cell derived exosomes may also reprogram immune cells to improve cardiac inflammation, wound healing and resolution (Figure 2).

## Circulating Exosomes and Sepsis in Heart Disease

The idea of plasma/circulating exosomes and their protective role in cardiac ischemia reperfusion injury (IR injury) arose from remote ischemia reperfusion studies. In experimental models, remote ischemia conditioning (RIC) reduced infarct size and protected against cardiomyocyte death post-MI. Molecular analysis showed that RIC increased HSP70 enriched micro vesicle release into the circulation. HSP70 protected against cardiomyocyte death via activation of ERK1/2, AKT and HSP27 through TLR4 (toll like receptor) signaling (Vicencio et al., 2015). Likewise, RIC in rats increased exosome associated miR-24 in rat plasma and protected against IR injury-induced cardiomyocyte death. Moreover, RIPC (remote ischemia pre-conditioning) exosome therapy reduced cardiomyocyte death, decrease infarct size and restored cardiac functions post-MI in rats (Minghua et al., 2018). Contradictory results have also been reported using RIPC in the heart, so future studies are essential to elucidate the mechanisms involved.

Circulating exosomes have also been implicated in sepsis mediated heart failure. Sepsis is dysregulated body’s response to infection, and is the leading cause of death in intensive care units (Raeven et al., 2018). Sepsis is primarily mediated by overactive inflammation, a process that leads to organ failure. While, a proper understanding of the molecular mechanisms surrounding sepsis is incomplete, recent studies have shown that circulating exosomes may partly explain the molecular mechanisms for sepsis induced cardiac pathology. Circulating exosomes increase with the onset of sepsis and are associated with progress of the disease (Terrasini and Lionetti, 2017); and so have been extensively investigated for diagnosis (Reviewed in detail elsewhere Raeven et al., 2018). Interestingly platelet derived exosomes in response to LPS induce vascular muscle cell death *in vitro* (Janiszewski et al., 2004). Likewise, circulating exosomes from septic patients inhibited myocardial contractility in isolated rabbit heart preparations, and these responses grew worse with prior exposure to LPS. Consistent with this, exosomes from septic patients inhibit contractions in papillary muscle preparation from rats. Interestingly, cardiac contractions were partially rescued through treatment with apocyanin, a Nox inhibitor. There was an increase in production of NO from septic exosomes that were inhibited by L-NAME, suggesting that NO could be the mediator of cardiac dysfunction in sepsis (Azevedo et al., 2007). Evidence for the involvement of exosomes in sepsis was further strengthened by an elegant study where pretreatment with GW4869 (inhibitor of exosome biosynthesis) protected against CLP (colon ligation and puncture) and LPS models of sepsis by reducing inflammation, improving cardiac function, and prolonging animal survival (Essandoh et al., 2015). Consistent with this report exosomes derived from sepsis mouse models induced disruptions of membrane podosomes, podosome cluster formation and increased vascular permeability (Mu et al., 2018). However, an interesting study by Gao et al. revealed exosomes packed with inflammatory mediators peaked 24 h after sepsis that induced proliferation of lymphocytes and differentiation of Th1, Th2 cells. Moreover, pretreatment of mice with these exosomes reduced inflammation, tissue injury, and improved survival in CLP model of sepsis (Gao et al., 2019). In light of these contradicting studies it remains to be seen whether GW4869 also inhibits synthesis of inflammatory mediators, or prevents peroxidation of membrane lipids as these are source of tissue damage. Studies have identified several microRNAs that are differentially expressed in the exosomes from septic shock patients, with very high levels of pro- inflammatory microRNA compared to exosomes from control patients. In addition, the patients that survived septic shock had high levels of microRNA involved in cell cycle regulation (Raeven et al., 2018), suggesting that exosomal microRNA has different functions at different stages of sepsis and can determine the pathogenesis and prognosis of septic patients.

These studies suggest that under healthy conditions, the body produces cardio protective exosomes that could be lost or altered under different metabolic settings or co-morbidities. Future studies would identify the source of these exosomes, characterize the cargo, and to design therapies targeting exosomal bioactive molecules.

## Cardiomyocyte and Cardiac Fibroblasts Affecting Cardiac Physiology

Cardiac remodeling is a classical response to various pathophysiological stressors such as increased peripheral resistance, arterial stenosis, heart failure and myocardial infarction (MI). The classical pathways involving the neuroendocrine system are well known. However, the molecular mechanisms involving exosomes have been instrumental in understanding the pathogenesis of these diseases. Exosomes released from different cell types within the heart could serve as intercellular communicators and influence cellular functions within the heart and in peripheral organs (Figure 1). The composition of the exosome cargo from cardiac tissue is determined by cardiac physiology, which can convey coded messages to the target cells and reprogram their biology. For example, exosomes released from cardiomyocytes under osmotic stretches and pressure overload are enriched in angiotensin type II receptor, and these exosomes were shown to induce vascular pressure changes in heart, muscle, and intestinal vessels (Pironti et al., 2015). Interestingly, exosomes from pericardial fluid surrounding the heart were enriched in miR-let-7b-5p and were shown to induce proliferation and vascular tube formation in endothelial cells, and restore blood flow in ischemic limb models through miR-let7b-5p (Beltrami et al., 2017). This data suggests pericardial fluid exosomes promote angiogenesis. Consistent with this, exosomes from heart explants from healthy individuals and heart failure patients had opposing effects in mouse models of MI (Qiao et al., 2019). Intracardiac injections of exosomes from explants of heart failure patients had worse outcomes in terms of heart function and cardiac remodeling when compared to exosomes from healthy explants in mouse models of MI, suggesting that exosomes from healthy and failing hearts were different. Molecular analysis revealed that exosomes from healthy hearts were enriched with miR-21 that inhibited apoptosis, promoted proliferation of cardiac cells, and promoted angiogenesis. At the molecular level, miR-21-5-p inhibited PTEN, and BCL2, and activated AKT and VEGF pathways in cardiomyocytes and endothelial cells (Qiao et al., 2019).

**FIGURE 3:**
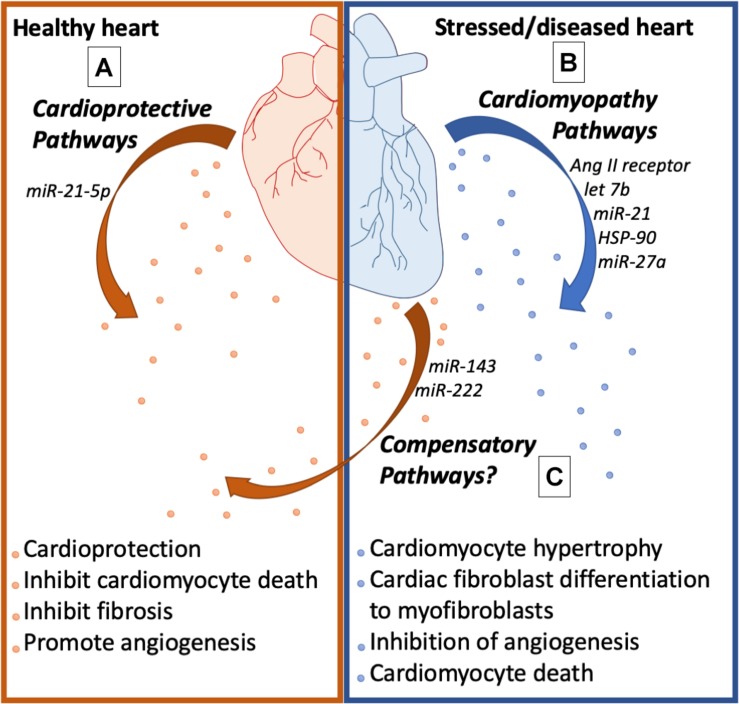
Cardiac health affects exosome biology. **(A)** Exosomes from the healthy heart are enriched with cardioprotective molecules that promote angiogenesis, inhibit fibrosis, and inhibit cardiomyocyte cell death. **(B)** Exosomes from the stressed heart release exosomes enriched with molecules that cause cardiomyocyte hypertrophy, myofibroblast differentiation, endothelial dysfunction, and cardiomyocyte cell death. **(C)** The stressed heart also releases exosomes that activate compensatory pathways to promote cardiac health.

Cardiomyocyte hypertrophy was induced in co-culture experiments using cardiac fibroblasts and cardiomyocytes (Fredj et al., 2005). In addition, conditioned media from cardiac fibroblasts induced cardiomyocyte hypertrophy, increased expression of vimentin and inhibited chronotropic contraction of cardiomyocytes *in vitro* (LaFramboise et al., 2007), suggesting that fibroblast derived factors were responsible for the phenotype. Interestingly, these factors were discovered to be exosomes derived from cardiac fibroblast (Bang et al., 2014; Lyu et al., 2015). Bang et al. have shown that these exosomes were rich in miR-21 and mediated cardiomyocyte hypertrophy through the inhibition of SORS2I and PDIM5, factors that are important for the prevention of hypertrophy. Consistent with this report, cardiac hypertrophic patients had high amounts of miR-21^∗^ in their pericardial fluid exosomes, and injection of these exosomes in murine models induced cardiac hypertrophy which was then shown to be inhibited by antago-miR-21 treatment. In addition, in angiotensin-induced cardiac hypertrophy, Lyu et al. identified that cardiac fibroblasts released exosomes that were enriched with renin, angiotensin, angiotensin receptors I, II and inhibited the angiotensin converting enzyme (ACR). At the molecular level, cardiac hypertrophy was mediated by the activation of RAS through the MAP kinase pathway (Lyu et al., 2015). These two studies have provided mechanistic insights into how cardiac fibroblast derived exosomes trigger pressure overload-induced cardiac remodeling. Cardiac hypertrophy also involves the differentiation of cardiac fibroblasts into myofibroblasts and increased synthesis of extracellular matrix proteins including collagen and MMP’s that result in cardiac fibrosis. Interestingly, studies by Datta et al. have uncovered that fibroblasts are reprogrammed by exosomes arising from cardiomyocytes. Cardiomyocyte specific HSP90 knockdown protected against fibrosis, hypertrophy, and cardiac dysfunction in renal artery ligation models of cardiac hypertrophy. In addition, conditioned media from hypertrophied cardiomyocytes induced collagen synthesis in cardiac fibroblasts that were inhibited through treatment with IL-6 neutralizing antibodies, suggesting the involvement of exosome mediated signaling (Datta et al., 2017). In addition, HSP90 increased exosome secretions that were enriched with IL-6 through p65 in cardiomyocytes that were involved in the programming of cardiac fibroblasts (Datta et al., 2017). Cardiac fibrosis and remodeling is also mediated by exosome derived miR-208 from cardiomyocytes post-MI. MiR-208 was enriched in exosomes from cardiomyocytes in response to hypoxia, angiotensin treatment and MI, that induces proliferation of cardiac fibroblasts; and inhibition of miR-208 using antagomiR protected against cardiac fibrosis and cardiac dysfunction post-MI. Exosome derived miR-208 inhibited Dyrk (dual specific tyrosine phosphorylated kinase) expression that phosphorylated NFAT to promote its nuclear export. Nuclear NFAT triggers fibrosis by inducing fibrogenic gene expression in cardiac fibroblasts. Moreover, injection of exosomes derived from the ischemic heart induced cardiac fibrosis in healthy mice (Yang et al., 2018). Likewise, miR-217 was enriched in cardiomyocyte derived exosomes in heart failure patients, thoracic aortic constriction models of heart failure and was involved in fibroblast proliferation and cardiac hypertrophy through PTEN (Nie et al., 2018). Consistent with this notion, cardiomyocyte-derived exosomes in response to isoproterenol and phenylephrine treatment were enriched with miR-300c, which inhibited angiogenesis by programing endothelial cells. In addition, treatment with antagomiR-300c protected mice against transverse aortic constriction induced cardiac hypertrophy (Ottaviani et al., 2018). Contrary to this are reports suggesting that cardiomyocyte-derived exosomes post-MI also induce angiogenesis by activating endothelial cells (Ribeiro-Rodrigues et al., 2017). Exosomes derived from H9C2 cells exposed to ischemic conditions induced endothelial cell proliferation, migration and tube formation. In addition, ischemia derived exosomes induced angiogenesis in CAM (chorio-allantoic membrane) assay using chicken embryos and Matrigel implants in rats. Furthermore, ischemic exosomes reduced infarct size, increased vascularization, and reduced fibrosis. At the molecular level, ischemic exosomes were enriched with miR-143 and miR-222, which both induce angiogenesis. In light of these contradicting reports, the speculation is that compensatory and decompensatory pathways were in action (Ribeiro-Rodrigues et al., 2017). Therefore, exosome content could be determined by the stage of cardiac pathology. In the early stages, cardiac exosomes could be enriched with cardio protective cargo and in terminal stages exosomes might be enriched with decompensatory cargo (Figure 3).

Oxidative stress is one of the primary causes of cardiomyocyte death and heart failure, post-MI. Interestingly, cardiac-derived exosomes post-MI are responsible for the loss of the cardiac endogenous antioxidant NRF2 (Nuclear factor erythroid-2 related factor) pathway. Ventricular expression of NRF2 was inhibited whereas the expression of miR-27a, miR-28-3p, and miR-34a were upregulated, post-MI. Moreover, TNF-alpha treatment in cardiomyocytes (CM) and cardiac fibroblasts (CF) increased expression and enrichment of these microRNAs in exosomes, suggesting that CM and CF secreted these exosomes post-MI. At the molecular level exosome derived miR-27a, miR-28-3p, and miR-34-a inhibited expression of NRF2 at the transcription level (Tian et al., 2018). Exosomes also mediate progenitor cell mobilization from bone marrow that play critical role in cardiac inflammation and remodeling. Injection of exosomes from mice post-MI, also increased the circulation of bone marrow-derived progenitors in systemic circulation. Exosomes from MI mice were enriched with myo-miR-1a, miR-208a, miR-133a, miR-499-5p that were taken up by BM progenitor cells and inhibited CXCR4 to promote their mobilization into the systemic circulation (Cheng et al., 2019).

## Cardiac Telocytes-Derived Exosomes

Telocytes are interstitial, cajal like cells present in the cardiac interstitium that have long extensions (telopods) that forms supportive frame work in organs (Cretoiu et al., 2016; Yang et al., 2017). Interestingly, telocytes have been found in the niches of stem cells and are known to support their growth (Cismas̨iu and Popescu, 2015). Telocytes are also involved in the horizontal transfer of macromolecules through exosomes (Mandache et al., 2007; Fertig et al., 2014; Cismas̨iu and Popescu, 2015; Cretoiu et al., 2016). Exosomes from telocytes are known to induce proliferation of vascular smooth muscle cells. Additionally, telocytes induced proliferation and migration of endothelial cells in *in vitro* in co-culture experiments, suggesting molecules released from telocytes were responsible. Moreover, telocyte derived exosome treatment protected rats from adverse effects of MI leading to dysfunction as shown by echocardiography and cardiac fibrosis (Yang et al., 2017). The data suggests that telocytes are unique in regards to providing niches to stem cells, and that their exosomes have protective effects in heart diseases. Future studies are needed to investigate if telocytes can be programmed *in situ* to protect from heart diseases.

## Exosomes From Cardiac and Peripheral Endothelial Cells

Endothelial cells (ECs) are the innermost monolayer of vasculature that lines the entire circulatory system including the heart. The endothelium regulates the normal vascular tone and permeability, maintains homeostasis, prevents thrombogenesis, and facilitates molecular exchange between circulating blood and vessel walls (Vane et al., 1990; Rajendran et al., 2013; Cahill and Redmond, 2016). Endothelium regulate these functions though the release of mediators such as adhesion molecules, cytokines, vasodilators, and exosomes into both the local tissues and systemic circulation (Vane et al., 1990; Galley and Webster, 2004; Sandoo et al., 2010). The exosome trafficking between ECs and cardiac tissue has been gaining attention due to exosomes’ ability to carry cardioprotective and proangiogenic factors to the heart. Exosomes from endothelial cells crosstalk with cardiomyocytes and other cardiac-resident cells, and are involved in many pathophysiological processes. Studies involving atherosclerosis, one of the common underlying causes of myocardial infarction, has found that communication between ECs and smooth muscle cells plays a crucial role in mediating protection against atherosclerosis development (Hergenreider et al., 2012). Hergenreider et al. showed that physiological shear stress robustly induces enrichment of atheroprotective and vasculoprotective microRNAs (such as miR-143/145) in endothelial exosomes, which when transferred to smooth muscle cells (SMCs) prevents SMC de-differentiation and induces an atheroprotective phenotype (Cordes et al., 2009; Hergenreider et al., 2012). Recently, it has been shown that exosomes derived from ECs help protect cardiomyocytes against ischemia and reperfusion conditions in AMI (Acute Myocardial infarction) by activating the ERK1/2 and MAPK signaling pathways (Davidson et al., 2018a). Another mechanism through which ECs help prevent cardiac remodeling in ischemic heart is through release of miR-126 and miR-210 enriched exosomes (Ong et al., 2014), which are well-known for activating pro-survival kinases and reducing cellular damage in recipient cells (Pan and Johnstone, 1983; Zaccagnini et al., 2017). Cardioprotective property of EC-derived exosomes were also shown in other cardiac diseases. Halkein et al. (2013) showed that in peripartum cardiomyopathy (PPCM), or pregnancy-associated heart failure, the delivery of miR-146a-enriched exosomes from ECs to CMs is increased. These miR-146a then reduce metabolic activity in CMs by decreasing the expression of N-Ras, Erbb4, Notch1, and Irak1, leading to cardiac dysfunction. Akbar et al. (2017) quantified and analyzed plasma exosomes in humans and mice after MI and found significant increases in EC-origin exosomes compared to non-AMI group (2.3–13.7-fold in human, 2.5-fold in mice) (Akbar et al., 2017). However, the author could not conclude if this increment is due to the cardiac ECs response to the MI, or the whole-body response due to activation of immune system after MI. The limitation of this paper and other studies (summarized in Table 3) highlights the lack of tools to determine tissue and cell specificity of exosomes in an *in vivo* setting. It is well known that ECs from different organs vary in terms of morphology, gene expression, growth factors, angiogenic potential and regulatory function; making it reasonable to believe that under a given pathophysiologic condition, the profile of exosomes from cardiac endothelial cells, the peripheral endothelium, coronary endothelium, or large vessels would differ. While exosome trafficking between cardiac ECs and cardiomyocytes is gaining attention, many of these studies have relied on HUVECs as the model instead of the targeted organ-specific ECs (such as cardiac microvascular ECs), therefore not truly mimicking the physiologic condition. Interestingly, several studies focused on non-cardiac pathology, in which cardiac EC are not the specific target, also imply the cardiac-protective effect of EC-induced exosomes. For example, study by Shyu et al. showed that under hyperbaric oxygen (HBO) therapy, human coronary artery ECs (HCAECs) can release exosomes containing long noncoding RNA MALAT1 (a proangiogenic RNA) into the circulation to reach the ECs in ischemic tissues, ultimately accelerating neovascularization and enhance wound healing process (Shyu et al., 2019). This explains the mechanism through which HBO reduce infarct size in MI *in vivo* (Sterling et al., 1993; de Jong et al., 2012; van Balkom et al., 2013). Other studies, for instance, have been carried out to investigate the role of miRNA and RNA in the exosome-mediated crosstalk between ECs, which can enhance angiogenesis in the injured tissue, as seen in the case of MI, hypoxia, inflammation or hyperglycemia. However, the direct role of peripheral endothelium-derived exosomes in cardiac pathophysiology remains poorly understood.

**TABLE 3:** Studies demonstrating endothelial cell-derived exosomes in pathophysiology of heart diseases.

**Donor cell**	**Target cell**	**Diseases**	**Model**	**Effect**	**References**
HUVECs	Cardiomyocytes (CMs)	MI	*In vitro*	HUVEC-derived exosomes protect CMs by activating the ERK1/2 MAPK signaling pathway	Davidson et al., 2018b
HUVECs	SMCs	Atherosclerosis (cause of MI)	*In vitro*	Under shear stress, HUVECs release exosomes containing atheroprotective and vasculoprotective microRNAs	Hergenreider et al., 2012
ECs	Cardiac progenitorcells (CPC)	Ischemia	*In vitro*	Overexpression of HIF1 (stimulation of hypoxic condition) in ECs leads to releasing of miR-126/210 enriched exosomes to reduce cellular damage in recipient cells	Ong et al., 2014
HUVECs	CMs	Peripartum cardiomyopathy (PPCM)	*In vitro*	The delivery of miR-146a-enriched exosome from ECs to CMs reduces metabolic activity in CMs by decreasing the expression of Erbb4, Notch1, and Irak1	Halkein et al., 2013
ECs/HUVECs	Monocytes	MI	*In vivo*/ *In vitro*	After AMI, circulating EC-origin exosomes significant increased	Akbar et al., 2017
HUVECs	CMs	Diabetes/MI	*In vitro*	Under the hyperglycemic culture conditions, cardioprotective ability of HUVEC-derived exosomes is eliminated	Davidson et al., 2018a

## Exosome Proteins and Their Function

Exosomes are actively synthesized and secreted by the endolysosome pathway, which involves active sorting of its contents including proteins. Exosomal proteomics has been instrumental in identifying the protein constituents of exosomes. Exosomes are enriched with ESCRT proteins, Tetraspanins, RNA binding proteins, selective sorting proteins, and membrane proteins that aid in sorting, cargo selection and stabilization of RNA (Xu et al., 2016). Some of these proteins- CD9, CD63, and CD81 have been extensively used as markers for exosome characterization. Exosome also carry specific proteins determined by the physiology of the donor cell that are involved in intercellular communication with specific functions. For example, several cancer cells release exosome that help in immune evasion, immunosuppression and cancer metastasis (Wortzel et al., 2019). Likewise, exosome derived proteins also participate in the pathophysiology of heart disease such as diabetic cardiomyopathy, cardiac ischemia reperfusion injury and sepsis (summarized in Table 4). Interestingly, several exosome-derived proteins have been targeted in diagnosis of various diseases including cancer (Cazzoli et al., 2013), myocardial infarction, sepsis, metabolic syndromes and cardiovascular outcomes (Bei et al., 2017). Due to this, active loading of specific proteins into exosomes is being used to engineer therapeutic exosomes. Several techniques including fusing of exosome sorted proteins with protein/peptide of interest, over-expression of the protein in donor cell, protein modification, *in vitro* enrichment of protein of interest in exosomes have been investigated (Liu and Su, 2019). However, therapeutic value of such exosomes is yet to be realized in clinics. More comprehensive overview of tools and technological advances in exosomal proteomics are previously described elsewhere (Barrachina et al., 2019).

**TABLE 4:** Exosome derived proteins in pathophysiology of heart diseases.

**Functional component**	**Donor cell**	**Functional outcome**	**References**
HSP60	Cardiomyocyte	Cardiomyocyte death	Kim et al., 2009
Renin, angiotensin receptor, ACE	Cardiac fibroblasts	Cardiomyocyte hypertrophy	Lyu et al., 2015
Ang type-II receptor	Cardiomyocyte	Cardiomyocyte hypertrophy	Pironti et al., 2015
HSP20	Cardiomyocyte	Endothelial cell proliferation anti-oxidant	Wang et al., 2016, p. 20
Cystasin C, Serpin F2, Serpin G1, Cd14	NA	Cardiovascular events	Kanhai et al., 2013
Microparticles (circulating and endothelial)	Endothelial cells	Prognostic marker for heart failure	Nozaki et al., 2009; Bei et al., 2017; Zamani et al., 2019
HSP70	Endothelial	Inhibit cardiomyocyte death	Davidson et al., 2018b
HSP 90	Cardiomyocyte	Cardiac fibrosis	Datta et al., 2017
Arginase 1	NA	Inhibit endothelin dependent aortic relaxation	Zhang et al., 2018
Mst1	Endothelial cells	Cardiomyocyte death in diabetes	Hu et al., 2018
Dystrophin	C2C12 cells	Restoration of dystrophin in cardiomyocytes in MDX mouse hearts	Su et al., 2018
Lamp2b	Cardiosphere derived cells	Increased retention of exosomes in the heart	Mentkowski and Lang, 2019

## Stem/Cardiac Progenitor Cell-Derived Exosomes in Cardiac Regeneration

An important aspect of heart disease is that cardiomyocytes have a very limited regenerative ability and any injury that leads to cardiomyocyte death results in the replacement with other cell types with limited electrophysiology function and contractile ability. Therefore, stem cell-based therapies are gaining traction for cardiac regeneration. Interestingly, the protective effect of these cells has more recently been attributed to the exosomes release (Ibrahim et al., 2014). Exosomes from stem cells can prevent cardiomyocyte death, inflammation, fibrosis, and promote angiogenesis by exporting microRNA, proteins, and signaling molecules that have regenerative capacity (summarized in Table 5). Consistent with this, several studies have shown that exosomes derived from cardiac progenitors and cardiosphere-derived cells have cardio protective and regenerative effects (Barile et al., 2014; Milano et al., 2019). Exosomes derived from stem cell/cardiac progenitor cell have advantages over the cells in terms of size, ease of production, problem with differentiation to undesirable non-cardiac cells and tumor formation. Therefore, exosomes from stem cells/cardiac progenitor cells can provide promising therapeutics for cardiac regeneration in the near future.

**TABLE 5:** Stem cell/cardiac progenitor cell-derived exosomes for cardiac therapy.

**Donor cells**	**Functional component**	**Functional outcome**	**References**
Cardiac progenitor cells	miR-210, miR-132, miR-146a-3P, miR-310	Prevent cardiomyocyte death	Barile et al., 2014
Cardiosphere-derived cells	miR-146a	Promote Angiogenesis Inhibit cardiomyocyte proliferation	Ibrahim et al., 2014
Sca1 + stem cells	HSF1	Cardiomyocyte Protection against ischemic injury	Feng et al., 2014
Embryonic stem cells	miR-290-295 clusters (miR-294)	Cardiac progenitor cell proliferation	Khan et al., 2015
Cardiosphere-derived cells	Y RNA fragment	Cardioprotection against oxidative stress	Cambier et al., 2017
Cardiac progenitor cells	Pregnancy associated plasm protein, IGF	Inhibit cardiomyocyte death	Barile et al., 2018
Cardiac progenitor cells	Activation of Akt-mTOR	Inhibit cardiomyocyte death	Li et al., 2018, 2019b
Implanted cardiac progenitor cells	miR-378, miR-623, miR-941 miR-1256, miR-384, miR-525-3P, miR-315-5P, miR-1224	Improved EF Inhibit fibrosis Angiogenesis	Saha et al., 2019
Cardiac progenitor cells	miR-146a	Protection against doxorubicin induced cardiotoxicity	Milano et al., 2019
C2C12 cells	Dystrophin	Restoration of dystrophin in cardiomyocytes in MDX mouse hearts	Su et al., 2018
MSC	miR-21-5p	Cardiac contractility and expression of ca handling genes	Mayourian et al., 2018
Endothelial progenitor cells (EPC)	IL-10, miR-375	IL-10 deficiency impairs angiogenesis in ischemic heart	Yue et al., 2017

## Exosome Engineering for Therapeutics

As previously discussed, exosomes have been shown to have a significant impact, whether positive or negative, on human disease, and exosomes from different cell types have unique characteristics that could potentially affect these exosome-induced changes in the disease milieu. While exosomes derived from known cell types must be considered, the ability of exosome engineering to alter the disease state is of direct current interest and the focus of many studies. However, the use of naturally produced exosomes for this purpose has some limitations, as “organic” exosomes may be limited in their specificity as they are derived from the parent cell, and it may be difficult to ensure that the desired content is what is being transported (Li et al., 2019a). To address these challenges, engineered exosomes offer an opportunity to use inherent characteristics from various exosome sources, combined with novel approaches, to improve exosome specificity, content loading, and production for improved therapeutic potential (Yim et al., 2016; Luan et al., 2017).

One of the challenges in using exosomes for therapy is ensuring that the desired cells or organs receive the exosomal contents (Li et al., 2019a). The most considered idea to address this as of now is parent cell membrane modification. The most commonly used method to achieve this is through inserting a gene encoding for a target protein into the parent cell, wherein the exosomes secreted will contain the added surface protein. For example, Liang et al. employed a plasmid encoding for a CD63/ApoA1 fused gene, which was then delivered to the parent cells. This gene was then incorporated and expressed as a surface protein which allowed for specific binding to the target HepG2 cells, via the SR-B1 receptor (Liang et al., 2018). Copper-catalyzed azide alkyne cycloaddition (CLICK chemistry) has also been explored for the use of bioconjugation of surface proteins in exosomes, and it has been reported that these modifications do not impact exosome size or internalization time (Smyth et al., 2014). Along with these methods, some alternative strategies have shown promise, as seen in the work of Armstrong et al. in using noncovalent interactions to modify the EV membrane. However, this method has challenges, as the reaction must be tightly controlled to prevent exosome loss of function due to aggregation (Qi et al., 2016; Armstrong et al., 2017).

The loading of specific exosomal content is also a popular area of research for potential exosome therapy. Passive loading usually involves incubating the drug or molecule either directly with collected exosomes, or with donor cells that secrete exosomes laden with the target molecule (Luan et al., 2017). However, passive loading has limited efficiency, and relying on donor cell secretion will incorporate nonspecific cargo along with the molecule of interest, requiring additional separation and purification steps for quality control. To combat this, active loading methods being explored to selectively incorporate a target molecule into exosomes. Sonication is one method being explored, as shown by Kim et al. (2016) in their study. In this study, macrophage derived exosomes were loaded through sonicating the exosomes to disrupt the membrane, allowing for more efficient incorporation of their molecule of interest, PTX (Kim et al., 2016). While this method allows for high loading efficiency, it can adversely compromise the exosome membrane, so care must be taken in regards to the frequency and time employed. Extrusion, the loading of the molecule of interest and exosomes through a syringe based lipid extruder, is also a method employed for active loading (Antimisiaris et al., 2018). This method shares both the high efficiency seen in sonication, as well as the drawback of potential compromised exosome membranes post loading, so care must be taken to ensure viable vesicles post loading. Electroporation is the most common method for loading siRNA or miRNAs, opening small temporary pores in the exosome membrane through electric field application to disrupt the phospholipid bilayer (Faruqu et al., 2018). However this method, while highly efficient, risks aggregation of exosomal contents, and this must be taken into consideration for study (Johnsen et al., 2016).

For clinical use, a major challenge is to increase exosome production to the necessary numbers for human patients, as current methods of exosome production and isolation are limited by low yield. The most basic way to perform this is to increase flask size for static culture, as having more cells and media would lead to greater exosome production. However, this method is generally not cost effective and can become space prohibitive with larger scale. One possible solution to increase efficiency is to increase secretion of exosomes from the cells. One of the easiest ways to achieve this is the application of stress. Several studies have shown that the application of heat, altered pH, hypoxia, or nutrient deficiency to induce cell stress all significantly increase the release of exosomes (King et al., 2012; Logozzi et al., 2018; Zou et al., 2019). However, the vesicles released under stress have been shown to have very different content profiles compared to unstressed exosome content, therefore, this must be taken into consideration for any potential use in engineered exosome therapy (Liu and Su, 2019). In addition, several drugs have been shown to regulate exosome secretion, as seen in a study by Datta et al. where they identified a number of drugs that regulate exosome biogenesis. Sitafloxacin, Forskolin, SB218795, Fenoterol, Nitrefazole and Pentetrazol were all shown to activate exosome biogenesis; while Tipifarnib and Ketoconazole were shown to be significant inhibitors of exosome release (Datta et al., 2018). Another potential approach to improve exosome secretion is to employ three-dimensional matrix cell cultures. Extracellular matrix scaffolds allow for three-dimensional signaling of the cells in culture, which can promote cell growth and vesicle release, and lead to a 20-fold increase compared to 2-D culture (Haraszti et al., 2018; Phan et al., 2018). Artificial scaffolds hold much promise, as not only do they allow for three-dimensional signaling, but also can be modified to promote vesicle release or influence vesicle content, as seen in a study by Du et al. In this study, a nitric oxide releasing polymer scaffold was used to grow mesenchymal stem cells (MSCs), and exosome release was studied. It was revealed that proangiogenic content such as VEGF and miR-126 inside the exosomes was increased due to the nitric oxide stimulation, demonstrating the efficacy of this scaffold material for potential therapeutic use (Du et al., 2017). Finally, one of the most promising technologies for large scale exosome production is the use of bioreactors. In a protocol established by Farid et al., the use of basic bioreactor flasks allowed for sequential collection of exosome containing media without passaging cells, and allowed for collection of 6.99 ± 0.22 × 1012 exosomes/mL (Faruqu et al., 2018). Another study, performed by Haraszti et al., combined both bioreactor technology with 3-D scaffold culture to take advantage of the benefits from both systems. In their model, the addition of a bioreactor tangential flow system increased the exosome yield from mesenchymal stem cells sevenfold over the 3-D scaffold alone (Haraszti et al., 2018).

There are many challenges remaining for the use of engineered exosomes in therapy. There are still concerns surrounding off-target effects, difficulty with control of exosome content loading, and observation that current exosome isolation techniques present inherent limitations on scaling up exosome production (Antimisiaris et al., 2018; Phan et al., 2018; Li et al., 2019a). Despite these challenges, significant strides have been made and new solutions are being pursued, with new research exploring selective targeting and loading, while improved isolation techniques are being tested as of this writing. Exosome engineering is a very young field, showing much promise, and with further research and ingenuity solutions there is a strong possibility of their use for therapy in the near future.

## Conclusion

Heart disease is the leading cause of morbidity and mortality in humans, and due to cardiomyocyte’s limited regenerative ability, novel therapies are needed to treat heart diseases. Exosomes secreted by different cell types play critical roles in the pathophysiology of heart diseases, and their biology is determined by tissue homeostasis or disease state of the host tissue. Consistent with this, exosomes from healthy hearts were found to be cardioprotective, while exosomes from stressed hearts were involved in furthering the pathogenesis of heart disease. Moreover, exercise was found to promote the secretion of cardioprotective exosomes into the circulation in diabetic mice, suggesting lifestyle choices may influence exosome biology to promote health. In addition, exosomes released from cardiac cells regulate the mobilization of immune cells and progenitor cells from bone marrow that are involved in the resolution of inflammation, cardiac remodeling, and wound healing. Likewise, several studies have demonstrated that exosomes can be programmed to protect and promote cardiac regeneration. Due to their unique size, content, ease of handling, non-immunogenic properties and being non-cellular, exosomes are an attractive new avenue for therapy. Therefore, further studies are needed to understand the detailed biology of exosome in health and disease, and how they may be engineered to develop novel therapies against heart disease.

## Author Contributions

MP and PK conceived the study. MP, HL, JH, DA, and GS collected the material and wrote the manuscript. PK provided critical feedback and helped to shape the manuscript.

## Conflict of Interest

The authors declare that the research was conducted in the absence of any commercial or financial relationships that could be construed as a potential conflict of interest.
